# Field populations of native Indian honey bees from pesticide intensive agricultural landscape show signs of impaired olfaction

**DOI:** 10.1038/srep12504

**Published:** 2015-07-27

**Authors:** Priyadarshini Chakrabarti, Santanu Rana, Sreejata Bandopadhyay, Dattatraya G. Naik, Sagartirtha Sarkar, Parthiba Basu

**Affiliations:** 1 Department of Zoology, University of Calcutta, 35 Ballygunge Circular Road, Kolkata – 700019, India; 2 Centre for Pollination Studies, University of Calcutta, 35 Ballygunge Circular Road, Kolkata – 700019, India; 3 Agharkar Research Institute, G G Agarkar Road, Pune – 411004, India

## Abstract

Little information is available regarding the adverse effects of pesticides on natural honey bee populations. This study highlights the detrimental effects of pesticides on honey bee olfaction through behavioural studies, scanning electron microscopic imaging of antennal sensillae and confocal microscopic studies of honey bee brains for calcium ions on *Apis cerana*, a native Indian honey bee species. There was a significant decrease in proboscis extension response and biologically active free calcium ions and adverse changes in antennal sensillae in pesticide exposed field honey bee populations compared to morphometrically similar honey bees sampled from low/no pesticide sites. Controlled laboratory experiments corroborated these findings. This study reports for the first time the changes in antennal sensillae, expression of Calpain 1(an important calcium binding protein) and resting state free calcium in brains of honey bees exposed to pesticide stress.

Pesticide exposure can have direct effects on individual bees as well as indirect effects on entire colonies^1^ and has been cited as one of the potential causes for global honey bee loss through colony collapse disorder^2^,^3^. Loss of pollinators especially honey bees, may have adverse bearing on agricultural economy and may also affect wild crop diversity, crop production, food security and overall ecosystem stability^4^,^5^. Apart from population loss^6^,^7^, other adverse effects include induced oxidative stress^8^ and behavioral deformities e.g. learning and communication, homing, foraging success and neurophysiology^6^,^9^,^10^. However, critical information gap exists on the response of natural populations of native honey bee to multiple pesticide exposures in field conditions^8^,^11^.

Olfaction plays a crucial and determining role in nectar and pollen search, mating, alarm, defense, orientation, self-colony recognition and incorporation of all conducts within the honey bee hive^12^,^13^,^14^. Measurement of proboscis extension response/reflex (PER) has been used to identify the extent of memory acquisition and retention in honey bees and has also been greatly used to assess the effects of pesticides on honey bees^10^,^15^. Odor detection is facilitated by olfactory receptor neurons (ORNs) located below various sensillae^16^. Sensillae types and distribution in honey bee species are well described through Scanning Electron Microscopic (SEM) studies^17^,^18^,^19^. In order to comprehend the neuronal processes fundamental to olfactory learning, biophysical properties such as ion channel activity and calcium ion (Ca^2+^) in particular have also been reported within the neurons of the olfactory pathway in the honey bee brain^20^,^21^,^22^. Long term memory (LTM) formation and its role in effective olfaction is an important survival strategy of the honey bees. It has been reported that most of the molecules shown to be involved in LTM formation depend directly or indirectly on Ca^2+^ 22 and several studies consequently suggest that Ca^2+^ may be the preliminary trigger for LTM development^22^. Many studies have established a link between Ca^2+^ concentrations and memory formation and processing^21^,^22^,^23^,^24^ with the argument that Ca^2+^ crucially helps in the establishment of “long-term potentiation” (cellular phenomenon underlying LTM)^22^,^25^,^26^. Calcium imaging studies have helped to establish how olfactory information is initially coded in the antennal lobe^27^,^28^, the primary olfaction centre^16^ and lateral horn and the mushroom bodies called the higher-order olfactory centers^16^. Reports have shown that Calpain, an important calcium handling protein, also plays a significant role in memory formation in the honey bee brains^29^,^30^,^31^.

In the present paper we attempt to assess the impact of pesticides on olfaction of natural populations of *Apis cerana,* a native Indian honey bee, sampled across pesticide intensive agricultural landscapes in an Eastern Indian state of Odisha^8^. We compared the field samples of *A. cerana* from a high pesticide intensive cropping site with a low or no pesticide intensity site for PER, morphometry, sensilla number and distribution and availability of resting state free calcium (Ca^2+^). We also corroborated the findings with controlled laboratory experiments. As per the previous study^8^, the chosen field sites in Odisha are Panchalingeshwar (Lat. 21.43 ^o^N; Long. 86.75 ^o^E), the low intensity cropping site (LIC) and Jaleshwar (Lat. 21.82 ^o^N; Long. 87.22 ^o^E), the high intensity cropping site (HIC).

## Results

### Intensification sites

The field sites were chosen based on the study by Chakrabarti *et al.* 2014^8^. The high intensity cropping site (HIC) – the high pesticide use site – recorded higher pesticide residues in the soil samples as well as higher pesticide use by the farmers compared to the low intensity cropping site (LIC) – the site of low pesticide use (Supplementary Figure 1).

### Morphometry measurements

All the honey bee samples were found to be morphometrically similar.

**Figure 1:**
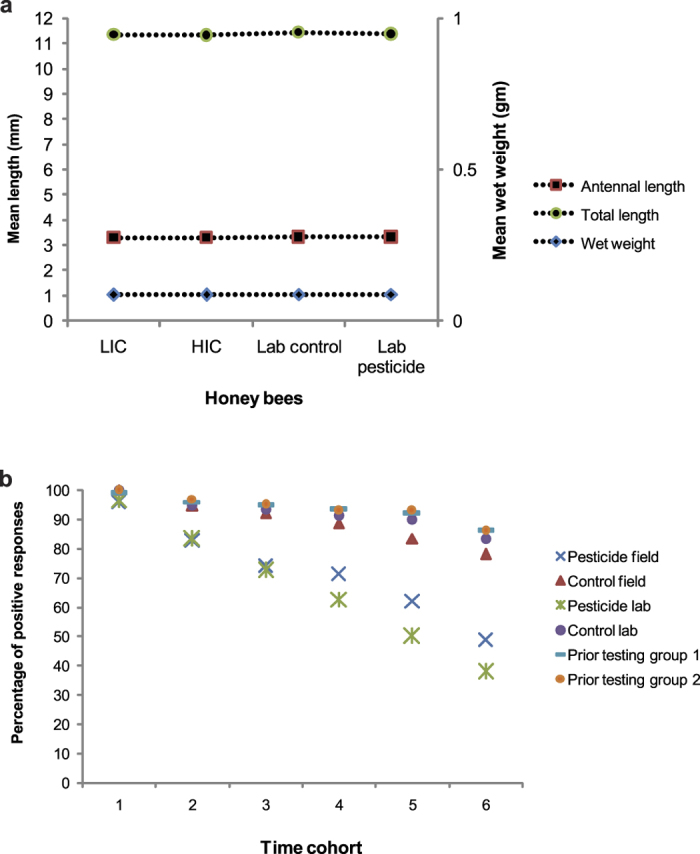
(**a**) Figure showing no significant difference in the honey bees with respect to the three morphometrical characters – antennal length, total length of body and wet weights – in all the groups of honey bees. Mean wet weight is plotted on the secondary axis. LIC is low intensity cropping site, HIC is high intensity cropping site, Lab control is the control group of honey bees in laboratory and Lab pesticide are the pesticide treated honey bees in the laboratory. (**b**) Figure showing significant decrease in proboscis extension response (PER) of honey bees treated with pesticides in laboratory and exposed to pesticides in field. There was no significant difference observed in PER of the honey bee groups prior to treatment in the laboratory.

No significant difference was observed between the honey bees sampled from LIC and HIC with respect to wet weight (p = 0.498, df = 298, t = 0.678), antennal length (p = 0.522, df = 298, t = 0.641) and total body length (p = 0.92, df = 298, t = 0.097). Mean total body length (8 X magnification), mean wet weight and mean antennal length (25 X magnification) of honey bees sampled from LIC were observed to be 11.32 mm ± 0.03, 0.085 gm ± 0.001 and 3.297 mm ± 0.01 respectively. Mean total body length (8 X magnification), mean wet weight and mean antennal length (25 X magnification) of honey bees sampled from HIC were observed to be 11.32 mm ± 0.04, 0.0846 gm ± 0.001 and 3.287 mm ± 0.011 respectively (Fig. 1a).

Similarly, no significant difference was also observed between the control and treatment groups of honey bee samples in the laboratory with respect to wet weight (p = 0.198, df = 298, t = −1.29), antennal length (p = 0.93, df = 298, t = 0.08) and total length of body (p = 0.09, df = 298, t = −1.68). The mean values of wet weight, antennal length (25 X magnification) and total body length (8 X magnification) in control groups were observed to be 0.0872 gm ± 0.0005, 3.3043 mm ± 0.009 and 11.438 mm ± 0.023 respectively and in treatment groups were found to be 0.086 gm ± 0.0009, 3.305 mm ± 0.008 and 11.365 mm ± 0.0371 respectively in the laboratory (Fig. 1a).

### Proboscis extension reflex (PER)

Proboscis extension reflex or response (PER) was checked in honey bees prior to testing in the laboratory. No significant difference was observed in PER across the six time cohorts between the two groups of honey bees – one group was then considered as control and the other was treated with pesticides (Table 1a). The positively responding honey bees were chosen for treatment experiments in the laboratory. The field samples were directly tested for PER. A significant decrease in PER in the honey bees sampled from HIC compared to LIC across all six time cohorts (1 minute, 3 minutes, 5 minutes, 10 minutes, 30 minutes and 60 minutes) (Table 1b) was observed. Similarly, a significant decrease was also observed in the treatment groups of honey bees in laboratory compared to their respective control groups across six time cohorts (Table 1b). This indicates that PER, being a direct behavioural response of olfactory learning and memory retention in honey bees, was affected in those honey bees which were exposed to/treated with pesticides in field and laboratory respectively (Fig. 1b).

### Scanning electron microscopic studies (SEM) of the honey bee antenna

**Figure 2:**
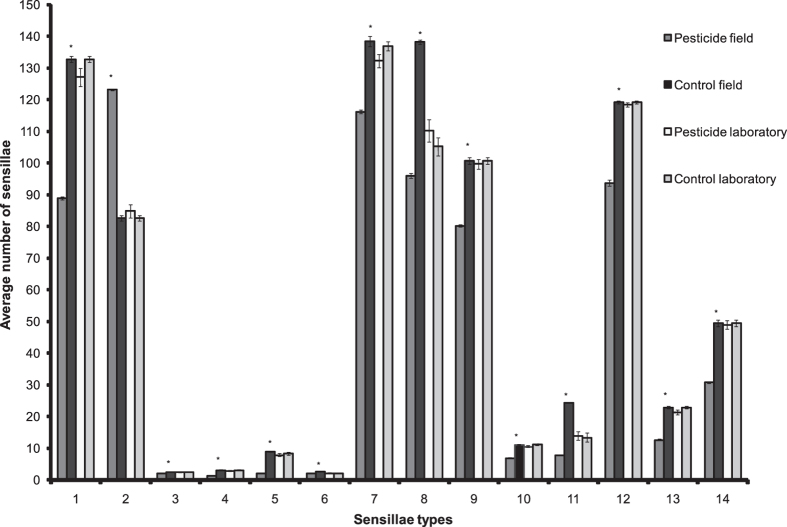
Figure showing mean number of antennal sensillae in all the honey bee experimental groups. 1: Sensory placodea, deep, 2: Sensory placodea, shallow, 3: Sensory ampullacea, 4: Sensory coeloconica, 5: Sensory basiconica, 6: Sensory campaniforme, 7: Sensory trichodea A, 8: Sensory trichodea B1, 9: Sensory trichodea B2, 10: Sensory trichodea C, 11: Sensory trichodea D, 12: Setae A1 & A2, 13: Seta A3, 14: Seta B. * = p < 0.05.

Having observed a direct behavioural response to pesticide, the anatomical changes in the honey bee antennae were then verified. The antennal sensillae of the honey bees, responsible for olfaction, were checked for abnormalities. A total of 14 sensillae types were identified across 10 antennal segments of the randomly collected foragers by SEM. Repeated measure ANOVA revealed a significantly higher number of different sensillae types, except one, in LIC (Supplementary Table 1). A significantly higher number in HIC was observed only with respect to sensory placodea shallow (Supplementary Table 1). However, no significant difference was observed in the populations exposed to pesticides in the laboratory after twenty four hours of pesticide exposure compared to the control groups (Supplementary Table 1). Tukey – Post Hoc test values with significance have been provided in Supplementary Table 2. The average number of sensillae in field and laboratory populations of honey bees for all the sensillae types is furnished in Fig. 2.

**Figure 3:**
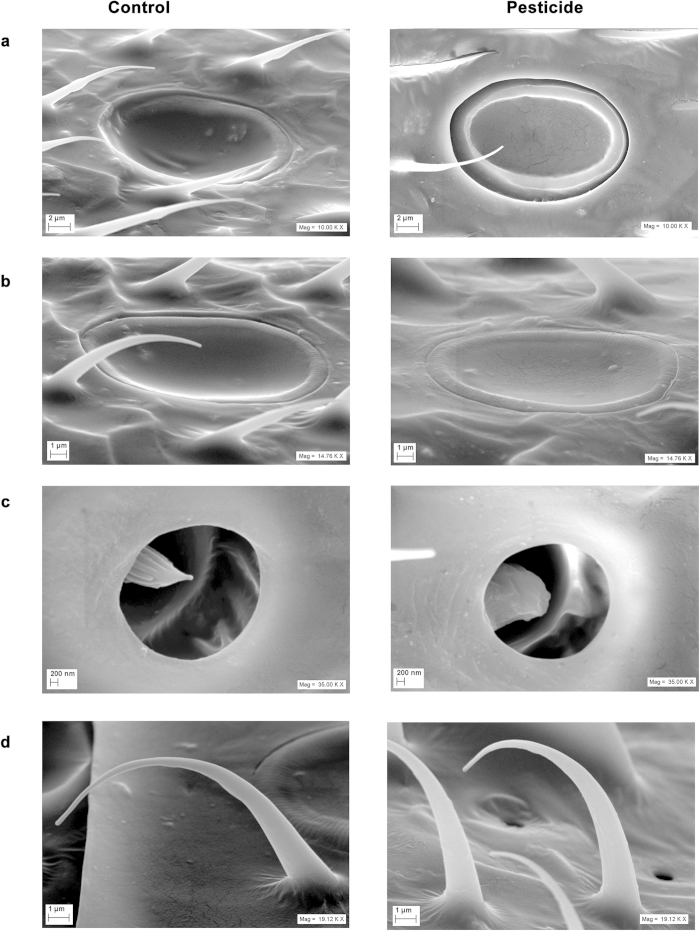
Figure showing representative scanning electron microscopic images of various antennal sensillae of honey bees across LIC (control) and HIC (pesticide) field sites. (**a**) Sensory placodea deep; (**b**) Sensory placodea shallow; (**c**) Sensory coeloconica; (**d**) Sensory trichodea B2. LIC is low intensity cropping site and HIC is high intensity cropping site.

A marked deformation was observed in the sensillae of the pesticide affected populations and in extreme cases, crack like marks were found on the sensory placodea deep, sensory placodea shallow (Fig. 3a,b) and sensory campaniforme (Supplementary Figure 2b) across various segments of the antennae. Sensory coeloconica seemed considerably deformed as well (Fig. 3c). A significant reduction in the diameter (p = 0.002, df = 58, t = 3.33) was observed in sensory campaniforme in HIC (16.18 μm ± 0.1) compared to LIC (16.72 μm ± 0.13). A significantly larger diameter (p = 0.0001, df = 58, t = 9.47) was observed in sensory placodea deep in LIC samples along the long axis (18.07 μm ± 0.12) compared to HIC samples (16.08 μm ± 0.18). The sensory trichodea B2 seemed considerably longer having more curvature in the control (LIC) populations of honey bees compared to HIC populations (Fig. 3d).

### Resting state free calcium availability in honey bee brains

The live honey bee brains were exposed by removing the cuticle and were stained with fura – 2 – dextran and tetramethylrhodamine dextran at the same time as the honey bee was stimulated with odour (linalool). The live brain cells had taken up the stain and the fluorescence observed was directly proportional to the resting state free Ca^+2^ of the stimulated live brain.

**Figure 4:**
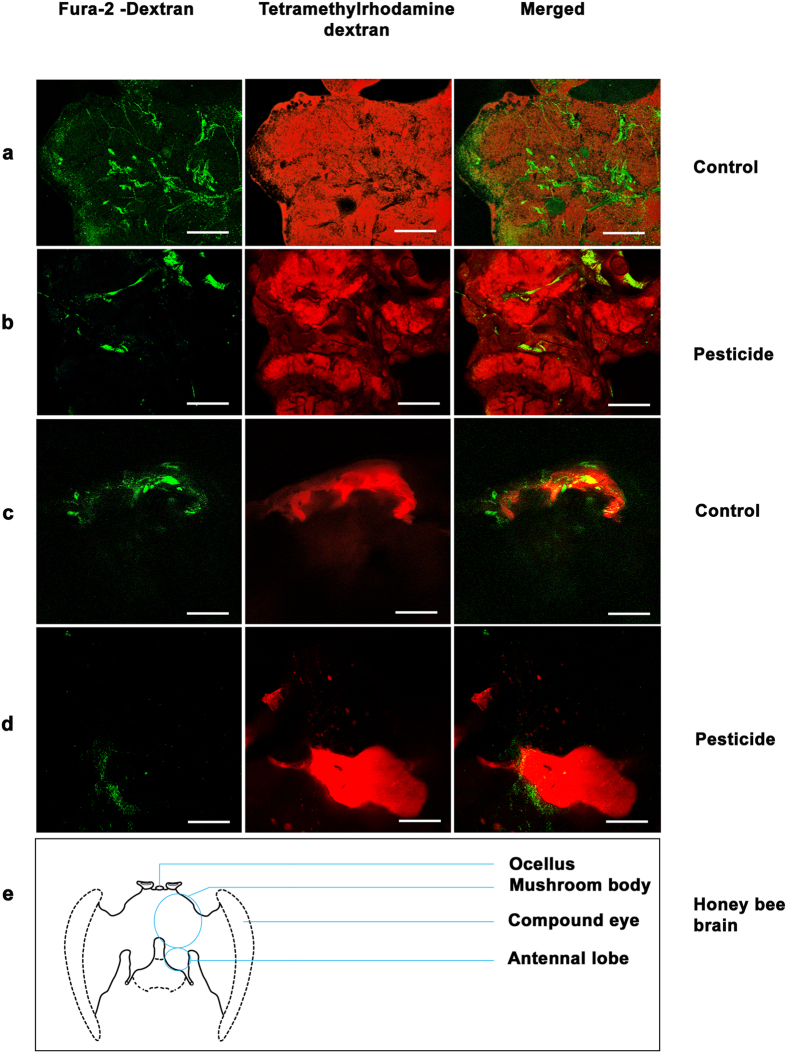
Figure showing olfactory regions of representative stained honey bee brain sampled from LIC (control) and HIC (pesticide) field sites. Magnification 20 X. Scale = 300 μm. Tetramethylrhodamine dextran stains whole brain red, Fura – 2 - dextran imparts green fluorescence and merged images show both stains together. (**a**) Part of mushroom body in LIC populations of honey bees; (**b**) Part of mushroom body in HIC populations of honey bees; (**c**) Antennal lobe region in LIC populations of honey bees; (**d**) Antennal lobe region in HIC populations of honey bees. LIC is low intensity cropping site and HIC is high intensity cropping site; (**e**) Line diagram of honey bee whole brain showing locations of mushroom body and antennal lobes.

**Figure 5:**
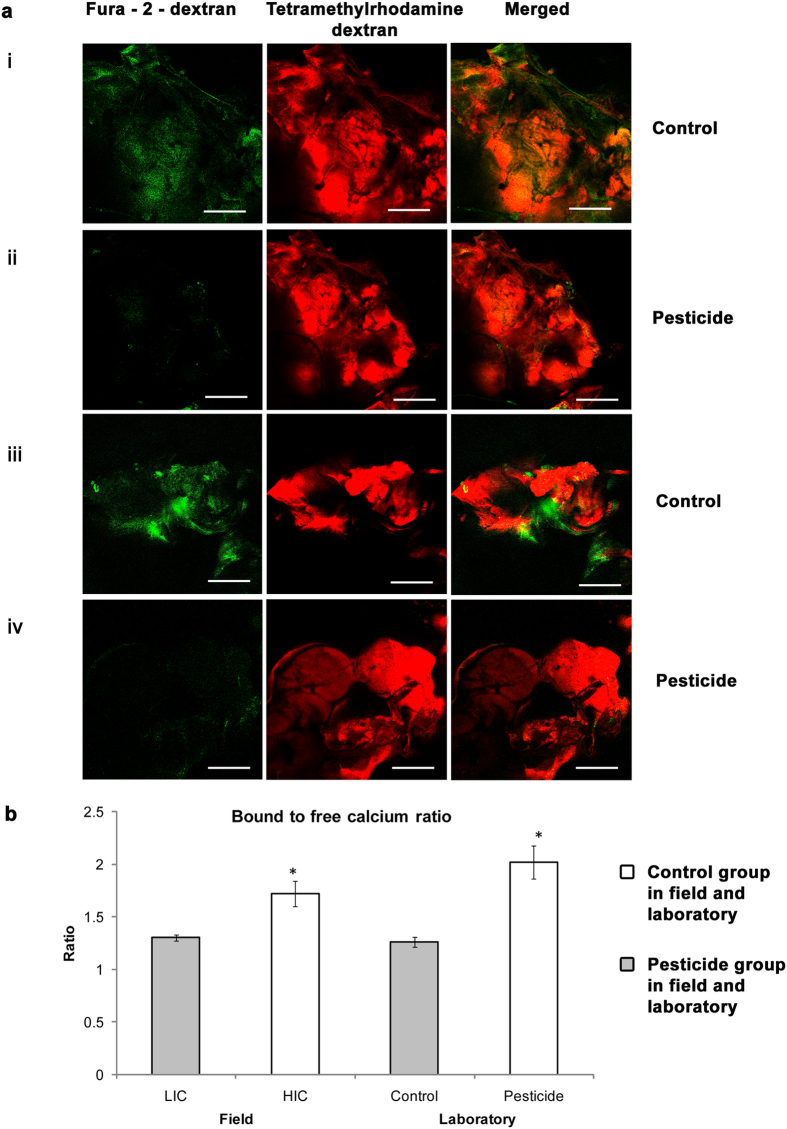
(**a**) Figure showing olfactory regions of representative stained honey bee brain sampled from control and pesticide groups in laboratory. Magnification 20 X. Scale = 300 μm. Tetramethylrhodamine dextran stains whole brain red, Fura – 2 - dextran imparts green fluorescence and merged images show both stains together. (i) Part of mushroom body in control groups of honey bees; (ii) Part of mushroom body in pesticide treatment groups of honey bees; (iii) Antennal lobe region in control groups of honey bees; (iv) Antennal lobe region in brains of pesticide treatment groups of honey bees. (**b**) Figure showing bound to free calcium ratios of honey bee populations across two field sites HIC and LIC and across laboratory groups of control and pesticide treated honey bees. LIC is low intensity cropping site and HIC is high intensity cropping site. * = p < 0.01.

A marked higher intensity of green fluorescence indicating free Ca^2+^ was observed in the brain (encompassing parts of mushroom body and the antennal lobe region of representative honey bee brains, stained with tetramethylrhodamine dextran and fura – 2 - dextran) of honey bees sampled from the LIC field sites compared to the HIC samples (Fig. 4). Similarly in the laboratory experiments, a lower fluorescence was observed in the mushroom body and antennal lobe regions of the brains of pesticide treated honey bees compared to the untreated control populations (Fig. 5a) indicating less free Ca^2+^ in the former group of honey bees.

Fluorimetry studies of the stained honey bee brains also revealed a significantly higher bound-to-free calcium ratio (z = −3.00227, p = 0.002680) for the HIC honey bees (1.81 ± 0.4) compared to the LIC samples (1.30 ± 0.1) (Fig. 5b). Similarly, a significantly higher bound-to-free calcium ratio (z = −3.26718, p = 0.001086) was observed in honey bees treated with pesticides in laboratory (2.02 ± 0.16) compared to untreated control group (1.26 ± 0.05) (Fig. 5b). A higher bound to free calcium ratio indicates less free Ca^2+^ in the brains of pesticide exposed/treated groups of honey bees and this corroborates with the fluorescence data from the confocal microscopic studies.

**Figure 6:**
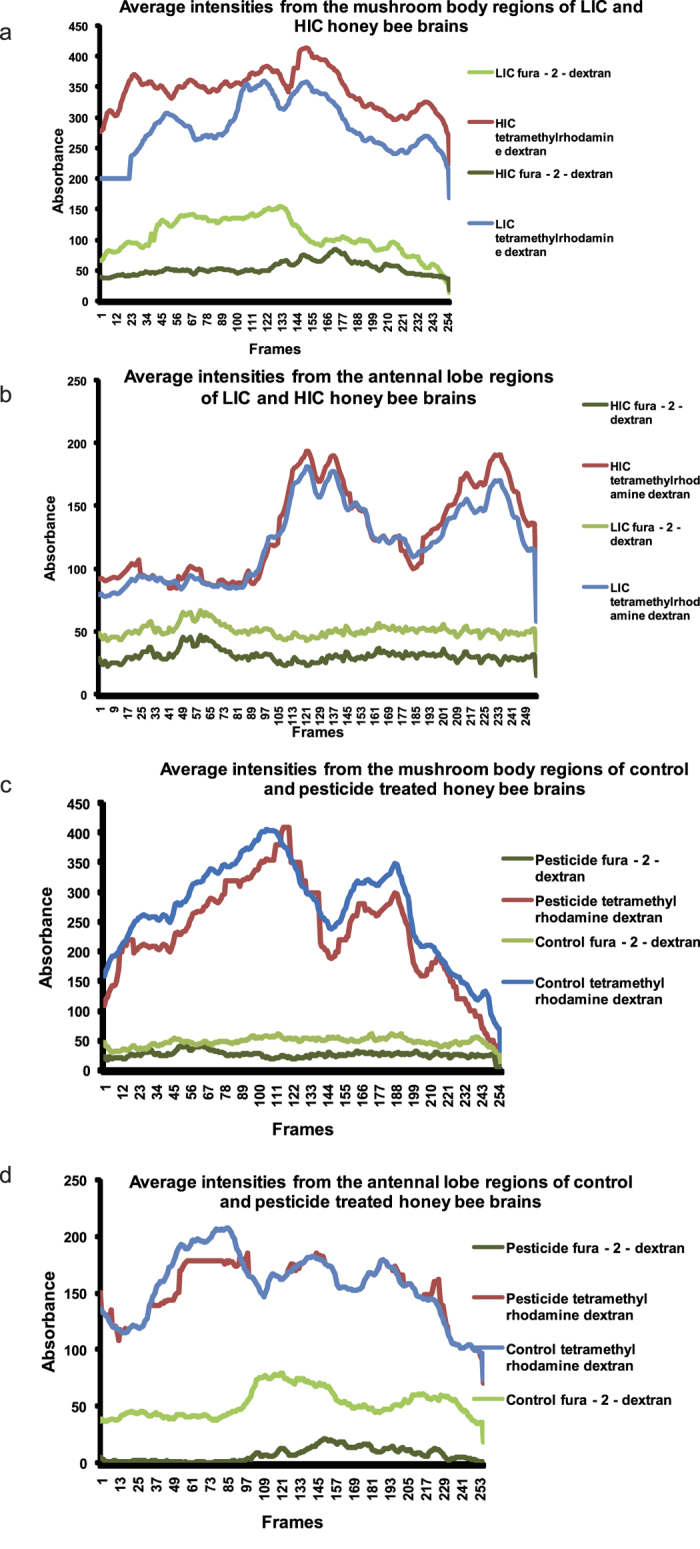
Figure showing the average intensities of two stains (fura – 2 – dextran and tetramethylrhodamine dextran) across the mushroom body and antennal lobe regions at every frame i.e. 4 μm optical sections up to 256 frames in two representative field samples of honey bees in (**a**) Mushroom body region and (**b**) Antennal lobe region. LIC: low intensity cropping or control site; HIC: high intensity cropping or pesticide site. Two representative laboratory samples of honey bees across the mushroom body and antennal lobe regions at every frame i.e. 4 μm optical sections up to 256 frames are shown in (**c**) Mushroom body region and (**d**) Antennal lobe region.

This was further corroborated by plotting the average intensities of both stains where a distinct down regulation was observed in fura – 2 – dextran absorbance in the field pesticide exposed honey bees (HIC) compared to LIC whereas tetramethylrhodamine dextran showed a similar trend between HIC and LIC (Fig. 6a,b). Figure 6a,b indicate the average intensities of both stains across the mushroom body and antennal lobe regions respectively in two representative field samples of honey bees (LIC and HIC) at every frame i.e. 4 μm optical sections up to 256 frames. Similarly, among laboratory samples, tetramethylrhodamine dextran also showed a similar trend between control and pesticide treatment groups whereas, a distinct down regulation was observed in the fura – 2 - dextran absorbance in pesticide treated samples compared to the control groups (Fig. 6c,d).

### Calpain 1 expression in honey bee brains

**Figure 7:**
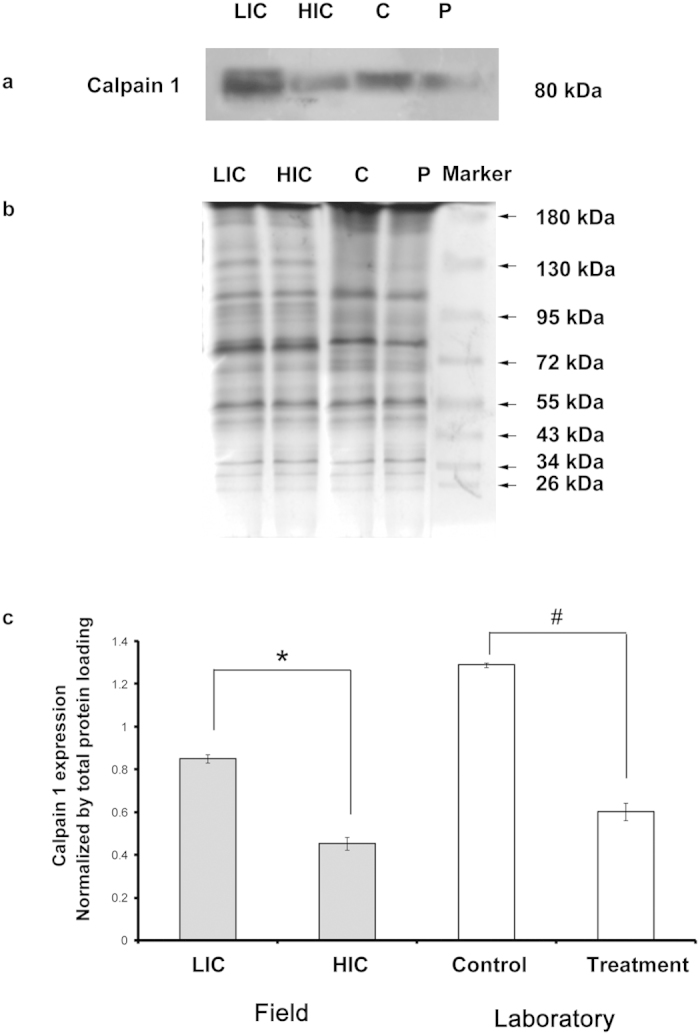
(**a**) Representative blots showing Calpain 1expression levels among honey bee brains from both field and laboratory populations; (**b**) respective SDS-PAGE gels stained with Coomassie blue confirming equal loading; (**c**) Graph indicates the change in expression of Calpain 1 normalized by Coomassie blue stained gel. C: Laboratory control and P: Laboratory pesticide treatment groups. LIC: Low intensity cropping site and HIC: High intensity cropping site. The marker lane shows the relative positioning of the bands. *p < 0.01 for field populations; #p < 0.01 for laboratory populations.

Western blot analyses revealed a significant difference in the expression of Calpain 1 in the brains of honey bees sampled across both LIC and HIC (1 tail Mann Whitney U test; n = 5) as well as in the laboratory. A significant increase of 1.87 ± 0.04 fold (1 tail Mann Whitney U test; n = 5; p < 0.01) was observed in Calpain 1 expression in LIC honey bee brain samples compared to HIC samples (Fig. 7). A similar response pattern was observed in the laboratory samples where, a significant increase in Calpain 1 expression (2.14 ± 0.07 fold), was observed in the brains of control honey bees compared to the pesticide treated individuals (1 tail Mann Whitney U test; n = 5; p < 0.01) (Fig. 7).

## Discussion

Multiple pesticide exposure for pollinators is a common phenomenon in intensive agricultural landscapes^8^,^32^. The increasing use of pesticides in developing countries^33^ has hence alarmingly raised the impacts of such pesticides on non target organisms. Although a number of laboratory studies have measured the impacts, it has been argued that laboratory studies of shorter durations are only a partial evaluation of the field realistic impacts^10^. Therefore, for a comprehensive assessment of the impact, field, semi – field and laboratory – all trials would be important in assessing such impacts^8^,^11^. In this context, our study holds added importance as we have not only verified that multiple pesticides induce impacts on the olfaction capacities of honeybees through laboratory trials, but also explored such impacts on wild populations of native honey bees.

The proboscis extension reflex (PER) is frequently used in a classical conditioning (Pavlovian) milieu for evaluating learning and memory in a variety of insect species including honey bees^34^. The ecological consequences of PER have been already reported^34^,^35^,^36^. It has also been reported through PER estimation that odour discrimination is frequently comparable between honeybees trained under controlled (honey bees in laboratory cages) and free-flying conditions (as in field populations of wild bees)^34^,^37^,^38^,^39^ and olfactory memories remain unchanged to variations in circumstances^34^,^40^,^41^,^42^,^43^, e.g., reallocation from natural to simulated environments^34^,^36^,^40^. Hence it can be assumed that in our study the significant changes in the observed PER between field samples is for differential pesticide exposure which is further corroborated by the laboratory experiments.

Since no significant difference was observed between the samples with respect to wet weight, antennal length and over all body length, we argue that difference in antennal sensillae in the randomly sampled foragers across two pesticide use sites was not because of any morphometric changes in the antennal characteristics across the gradient but because of pesticide exposure. The treatment and control samples from laboratory showed no significant difference in antennal sensillae after twenty four hours of exposure. We argue that the changes in sensillae in the honey bees exposed to pesticides happen over longer pesticide exposure in natural condition and can be attributed to developmental impairments leading to deformed/reduced number of sensillae. Pesticide exposure happens as contaminated food is brought back to the hive by the foragers and the subsequent honey bee generations are reared on it. Honey bees can be easily exposed to pesticides over long durations of time by transfer of such pesticide residues to the hives^44^. Our observed significant difference in the field populations’ sensillae and not in the shorter duration laboratory trials is due to the former’s much longer exposure to pesticides spanning several generations.

Even though a significant difference was observed in the sensillae numbers between LIC and HIC samples of field populations of honey bees, the average number of only sensory placodea shallow was higher in HIC populations compared to LIC populations unlike rest of the other sensillae. We assume that there may have been a gradual transformation of sensory placodea deep to sensory placodea shallow in the pesticide exposed populations. The observed affected sensillae in our study have been reported earlier to play an important role in olfaction^45^,^46^,^47^. Hence, their deformation, damage, size and number reduction in the antennae of pesticide exposed wild honey bees can be assumed to interfere with olfaction. To our knowledge, this is the first report of any change in the antennal sensillae in the pesticide exposed natural honey bee populations.

It was observed that the control populations in laboratory and the LIC field samples showed higher fluorescence in the honey bee brain, particularly around the antennal lobe and the mushroom body regions, and lower bound to free calcium ratio compared to their respective pesticide treatment groups and HIC samples. This is indicative of less free Ca^+2^ in pesticide exposed/treated honey bee brains and may affect the processes underlying olfaction which are dependent on availability of free Ca^+2^. The laboratory results have helped to validate our field results. A lower bound to free Ca^2+^ ratio indicates higher free Ca^+2^ and hence a higher fluorescence as evident from our results. It has been reported that only free Ca^2+^ is biologically active^48^. It is also known that Ca^2+^ indicators bind and interact only with freely diffusible Ca^2+^ ions^49^ and that free Ca^2+^ - fura-2 emits greater than bound Ca^2+^ - fura-2 when excitation wavelength is longer than 370 nm^50^. It was also observed that between HIC samples and pesticide treated laboratory samples, the HIC samples showed a higher fluorescence. We assume that this could be because in laboratory, the honey bees are only allowed to feed from the pesticide mixture. However in field, they still have an additional option – even though very limited in HIC as evident from our previous report of field surveys^8^ – to feed from less pesticide sprayed or pesticide free natural vegetation.

Calpain is an important calcium handling protein^30^. It cleaves protein kinase C (PKC) to its activated form protein kinase M (PKM) which in turn is responsible for memory formation^30^. Calpain has also been previously reported in the honey bees^29^ and is known to play an important role in memory formation^30^,^31^. Our study indicates a higher expression of Calpain 1 in brains of control populations of honey bees, from both field and laboratory. This may trigger a more efficient handling of free calcium which in turn might benefit the olfactory learning and memory in the honey bee population that are not exposed to pesticides. Changes in Calpain 1 expression level corroborated our findings of confocal microscopy and fluorimetric analyses for resting state free calcium during pesticide stress.

To our knowledge, this is also the first report of change in resting state free Ca^2+^ across honey bee populations due to pesticide exposure, as studied by confocal microscopy and fluorimetric analysis. These findings also show a decrease in resting state free Ca^+2^ and Calpain 1 expression in honey bee brains exposed to pesticides and to our knowledge, this is the first report of such phenomenon in natural honey bee populations in the intensive agricultural sites with high pesticide load.

Pollen odour cues help bees to locate food resources^51^. Enhanced olfactory learning performance helps to improve foraging success in bees, as evident from reports in bumble bees^52^ and honey bees^53^. Also, daily tasks of honey bees are set by pheromonal instructions^14^ in which olfaction, once more, is a crucial factor. Therefore, impaired olfaction would have strong influence on the population health. Pesticide treatment may lead to impairment in olfaction, memory or both. However the effects of pesticides on memory impairment alone will require further investigation. However, further investigation is necessary to check the dynamics of calcium flux in the honey bee brains exposed to pesticide load.

Our studies hence not only show the adverse effects of pesticide exposure on an important behaviour such as PER of the honey bees but also delve into the underlying micro structural and neuro-physiological processes contributing to such changes. Damaged olfaction thus might have critical consequences for the very survival of honey bees and such assessment requires to be undertaken for other non – target insect groups too especially for other beneficial pollinator groups.

## Materials and Methods

Detailed methods have been incorporated in supplementary information (supplementary methods).

### Agricultural intensification landscapes

The agricultural landscape was chosen in the Eastern Indian state of Odisha based on the work by Chakrabarti *et al.* (2014)^8^. Two locations chosen were marked as high intensity cropping (HIC) and low intensity cropping (LIC).

### Sampling honey bees

The individual foragers of *Apis cerana* were randomly sampled at the nest entrance. A total of three colonies in each site were chosen for all experiments.

### Exposure of honey bees to pesticides in laboratory and pesticide treatments

The honey bees were acclimatized and reared in laboratory cages based on the methods by Chakrabarti *et al.* 2014^8^. Three pesticides in combination were used as was reported in a previous study^8^ - an organophosphorus (OP) pesticide, a synthetic pyrethroid (SP) - and an endosulfan pesticide (ES) – in combination as 12.5% OP + 4% SP + 15% ES (as comparable to the doses used by farmers in field).

### Morphometric measurements

Randomly sampled forager honey bees from field sites were used for morphometric measurements of total body length, antennal length and wet body weights.

### Proboscis extension reflex (PER) studies

The protocol is based on the methods described by Bitterman *et al.* (1983)^54^, Sandoz *et al.* (2000)^41^, Decourtye *et al.* (2004)^55^, Decourtye *et al.* (2005)^9^, Frost *et al.* (2012)^34^ and Kirkerud *et al.* (2013)^56^. The source of odour was a small piece of filter paper (40 × 30 mm^2^) soaked in 10 μl of linalool (95–97% purity, Sigma, U.S.A.). Positive PER were recorded as “Yes” or “1” and negative responses were recorded as “No” or “0” during the test trials where only odour was delivered to the honey bees across 6 time cohorts.

### Scanning electron microscopy (SEM)

A total of 14 sensilla types were identified across 10 antennal segments of the randomly collected foragers^17^,^18^ by SEM.

### Calcium imaging using confocal microscopy

Live honey bees were mounted on a glass holder after anesthetizing them over ice. Low melting point hard wax was used to fix the eyes and thorax to the glass holder. The cuticle was gently removed from the head region and the stain concoction (fura – 2 – dextan and tetramethylrhodamine dextran) was injected in to the soma region of the mushroom body. The staining method was based on the modified protocol of Haehnel *et al.* (2009)^57^. The brains were removed and prepared for confocal microscopy^57^. Whole brains were mounted for confocal imaging^58^,^59^,^60^.

### Bound to free calcium ratio using fluorimetry

The absorbance or optical density (OD) values of bound to free calcium was calculated for the pesticide and control groups of honey bees from both field and laboratory treatment experiments by the formula^49^ Δ*Ca*^2+^ = *F*_*CaB*_ ÷ *F*_*CaF*_. Here ΔCa^2+^ is the ratio of bound to free calcium; F_Ca B_ and F_Ca F_ are the absorbance values of bound and free calcium respectively.

### Western blot and quantification of Calpain 1

Protein preparation, western blotting and band quantification were done based on previous studies^8^. Calpain 1 bands were obtained from thirty microgram of total protein extract from honey bee brains through western blotting technique. Equal loading of protein samples was confirmed by Coomassie blue staining of the gel. The blots were scanned; bands were normalized by Coomassie and quantitated using GelDoc XR system and Quantity One^®^ software version 4.6.3 (Bio-Rad, California, USA).

### Data Analyses

Data was analyzed using Statistica software (version 10).

## Additional Information

**How to cite this article**: Chakrabarti, P. *et al.* Field populations of native Indian honey bees from pesticide intensive agricultural landscape show signs of impaired olfaction. *Sci. Rep.* **5**, 12504; doi: 10.1038/srep12504 (2015).

## Supplementary Material

supplementary information

## Figures and Tables

**Table 1:** 

Time cohorts	**a. Prior to treatment**			**b. After treatment/Field samples**					
	**Laboratory**			**Field**			**Laboratory**		
	**p value**	**df**	**t value**	**p value**	**df**	**t value**	**p value**	**df**	**t value**
**1 minute**	0.318122	298	−1	0.0132	298	2.491653	0.024125	298	2.2667
**3 minute**	0.585547	298	0.545894	0.00098	298	3.3275	0.001633	298	−3.17897
**5 minute**	0.628186	298	0.484784	0.000027	298	4.260149	0.00001	298	4.971314
**10 minute**	0.516657	298	0.649281	0.000033	298	4.217457	0.00001	298	6.25347
**30 minute**	0.546585	298	0.603575	0.000002	298	4.875576	0.00001	298	8.373628
**60 minute**	0.523152	298	0.639249	0.00001	298	5.515639	0.00001	298	9.042558

(**a**) Table shows no significant differences in the proboscis extension responses of honey bees in laboratory prior to treatment across six time cohorts. (**b**) Table shows significant decrease in proboscis extension responses in honey bees exposed to/treated with pesticides in both field and laboratory samples compared to control groups of honey bees across six time cohorts.
