# Supplementary material for: Field populations of native Indian honey bees from pesticide intensive agricultural landscape show signs of impaired olfaction
Source: Sci Rep. 2015 Jul 27;5:12504. doi: 10.1038/srep12504 (PMC4648454; doi:10.1038/srep12504)

Field populations of native Indian honey bees from pesticide intensive agricultural landscape show signs of impaired olfaction.

Priyadarshini Chakrabarti [1,2], Santanu Rana [1], Sreejata Bandopadhyay [1], Dattatraya G. Naik [3], Sagartirtha Sarkar [1] and Parthiba Basu [1, 2] *

1. Department of Zoology, University of Calcutta, 35 Ballygunge Circular Road, Kolkata – 700019, India

2. Centre for Pollination Studies, University of Calcutta, 35 Ballygunge Circular Road, Kolkata – 700019, India

3. Agharkar Research Institute, G G Agarkar Road, Pune – 411004, India

* Corresponding author email: bparthib@gmail.com

Methods:

Agricultural intensification landscapes:

The agricultural landscape chosen is in the Eastern Indian state of Odisha. Two locations were chosen marked as high intensity cropping (HIC) and low intensity cropping (LIC) based on the work by Chakrabarti et al. (2014) [8].

The HIC site chosen for our study was Jaleshwar (Lat. 21.82 ^{o} N; Long. 87.22 ^{o} E) and the LIC site was Panchalingeshwar (Lat. 21.43 ^{o} N; Long. 86.75 ^{o} E). Pesticide residue analyses in soil and honey bee body have already further confirmed the establishment of the differential pesticide intensities of these study sites along with the cropping intensity data and farmer survey data of cropping and pesticide use [8].

Sampling honey bees:

The individual foragers of *Apis cerana* were randomly sampled at the nest entrance. A total of three colonies in each site were chosen for PER studies, morphometric measurements, SEM imaging, confocal microscopic studies for Ca^{2+} imaging and fluorimetric analyses.

Exposure of honey bees to pesticides in laboratory:

Fresh set of three colonies of *Apis cerana* from LIC site was brought to the laboratory and acclimatized in separate cages for two days before experimenting on them. The cages mimicked near field conditions as they were placed outdoor. The honey bees were fed ad libitum 3 molar sugar syrup and dry multifloral pollen[8]. The smaller experimental cages were similar to the protocol mentioned in Chakrabarti et al. (2014) [8].

They were exposed to the pesticides for 24 hours before performing any experiment on them. The bees were starved overnight before the experiment started. The feeder tubes were replaced after six hours with fresh sugar syrup and / or pesticide-treated sugar syrup. Mortality was checked and feed consumption was also recorded[8, 9].

Pesticide treatments:

Three pesticides in combination were used as was reported in a previous study [8] - an organophosphorus (OP) pesticide, a synthetic pyrethroid (SP) - and an endosulfan pesticide (ES).

From earlier studies [8], it was found that the treatment comparable to field dose and from which point significant changes were observed in experimental honey bee populations in laboratory is 12.5% OP + 4 % SP + 15% ES. Since the forager honey bees come in direct contact with pesticides in field, the treatments must be made comparable to the field dose [8]. The pesticide

combination used for mixture treatment was hence 12.5% OP + 4 % SP + 15% ES (as comparable to the doses used by farmers in field).

Morphometric measurements:

Randomly sampled forager honey bees from field sites were used for morphometric measurements using Olympus SZ61 stereozoom microscope (Olympus, USA) and Olympus oculometer under 8 X magnification. For this particular study, only the honey bee total body length, the antennal length and the honey bee wet body weights are considered. Wet weight was measured by help of Vibra HT, Essae, Japan. A total of 50 random foragers were selected from each of the three colonies in each of the two field sites as well from each of the three colonies in laboratory control and pesticide treated groups.

Proboscis extension reflex (PER) studies:

A total of 50 individuals were used for experiments per colony. The honey bees were starved for four hours prior to conditioning [9, 41]. The honey bees were individually mounted in plastic tubes with only their heads free i.e. antennae and mouth parts could move freely. The tubes were individually placed in glass box. The protocol is based on the methods described by Bitterman et al. (1983) [54], Sandoz et al. (2000) [41], Decourtye et al. (2004) [55], Decourtye et al. (2005) [9], Frost et al. (2012) [34] and Kirkerud et al. (2013) [56]. The source of odour was a small piece of filter paper (40 x 30 mm^2) soaked in 10 µl of linalool (95-97% purity, Sigma, U.S.A.) placed in a Pasteur pipette through which air was flowed in at a constant rate. A vacuum desiccator (Borosil, India) was used to remove the odorant air after each trial of conditioning for each bee.

Before the onset of PER trials, each bee was checked for intact PER by gently touching the antenna with 300 g L^{-1} sucrose solution. Only the responsive honey bees were considered for experiments. Before every trial, the bees were familiarized with the experimental set up. The odour stimulus was provided for 6 seconds, at 3 seconds of which the antenna was touched with 300 g L^{-1} sucrose solution. Before the odour stimulus ended, the bees were rewarded to similar concentration of sucrose solution. There were three conditioning trials, after which seven test trials were given to the honey bees at 1 min, 3 min, 5 min, 10 min, 30 min, 60 min and 120 min interval. Positive PER were recorded as "Yes" or "1" and negative responses were recorded as "No" or "0" during the test trials where only odour was delivered to the honey bees.

Scanning electron microscopy (SEM):

Random samples of 10 honey bees were taken from each of the three colonies in each LIC and HIC sites. The foragers were caught at the entrance of hives. Antennae were removed and refluxed in carbon tetrachloride following by 20 nm gold coating using Q150T ES sputter coater (Quorum Technology Ltd., UK) before SEM imaging was done by [17] ECO Special Edition (Carl Zeiss, Germany). The laboratory treated honey bees were also studied. A total of 14 sensilla types were identified across ten antennal segments of the randomly collected foragers[17] by SEM.

Calcium imaging using confocal microscopy:

In vivo preparation for calcium imaging was done on adult forager honey bees. The foragers were randomly sampled from the hive entrance. 10 honey bees were studied from each of three 3 colonies in each study site and laboratory groups. The staining method was based on the modified protocol of Haehnel et al. (2009) [57]. For honey bee brain staining, 10:1 mixture of fura-

2 dextran (Invitrogen, U.S.A.) and lysine fixable tetramethylrhodamine dextran (Invitrogen, U.S.A.) was used [57]. TMRD stains the whole brain while FD fluoresces in presence of Ca^{+2}. Live honey bees were mounted on a glass holder after anesthetizing them over ice. Low melting point hard wax was used to fix the eyes and thorax to the glass holder. The cuticle was gently removed from the head region and the stain concoction was injected in to the soma region of the mushroom body under Olympus SZ61 stereozoom microscope (Olympus, USA) all the while stimulating with linalool (as used in PER experiments). The cuticle piece was restored and the honey bees were allowed to recover for four hours at 20 ^{O} C in a humidified case. The brains were removed and prepared for confocal microscopy as described in the protocol by Haehnel et al. (2009)[57]. Whole brains were mounted for confocal imaging [58 – 60] using Olympus FV1200 confocal microscope (Olympus, USA).4 µm optical sections were viewed.

Bound to free calcium ratio using fluorimetry:

Honey bees were processed similarly with staining of brain for fluorimetric analyses as mentioned in previous section. However, only fura – 2 – dextran was used for fluorimetric studies. The brains were dissected out and tissue lysates were prepared by homogenization of tissue in 1X lysis buffer (50mM Tris-HCl, 250mM NaCl, 0.5% NP40, 10% glycerol and 0.5 % EDTA – free protease inhibitor cocktail (Roche, California, USA). Fura – 2 - dextran has excitation/emission spectra of 363 nm / 512 nm and 335 nm / 505 nm for free and bound calcium respectively [49–50]. However, for ratiometric measurements, 380 nm and 340 nm had been considered as the excitation spectra for free and bound calcium respectively [49-50]. The absorbance or optical density (OD) values of bound to free calcium was then calculated for the pesticide and control groups of honey bees from both field and laboratory treatment experiments by the

formula[49] $\Delta Ca^{2+} = F_{CaB} \div F_{CaF}$. Here ΔCa^{2+} is the ratio of bound to free calcium; F_{CaB} and F_{CaF} are the absorbance values of bound and free calcium respectively.

Three colonies were studied in each field site and also in each control and pesticide treatment groups in the laboratory. Three replicates were taken from each colony and each replicate had twenty individual honey bees where twenty live brains were stained, dissected out, lysates were prepared and the absorbance readings were taken immediately with the help of a Varioskan Flash microplate reader (Thermo Scientific, USA). Background fluorescence was subtracted by taking absorbance readings from unstained tissue samples.

Preparation of protein samples for western blot:

Protein sample preparation was based on the protocols of Chakrabarti et al. (2014) [8]. Whole honey bee brains were ground and samples were washed in chilled 1X PBS and were homogenized in protein extraction buffer pH 7.5 [50mM Tris-HCl, 250mM NaCl, 0.5% NP40, 10% glycerol and 0.5% EDTA – free protease inhibitor cocktail (Roche, California, USA)] using Dounce homogenizer (sigma, USA) for 5 minutes. The tissue lysates were then subjected to centrifugation at 14700 g at 4°C for 20 minutes. The supernatants were collected from each sample and concentrations of protein were estimated by Bradford assay (Bradford, 1976).

Western blot analysis for Calpain 1 expression:

Western blotting and band quantifications were done following the protocols of Chakrabarti et al. (2014) [8]. Thirty micro gram of total protein extract - from each of the field (LIC and HIC) as well as the laboratory samples (control and treatment)- was fractionated by SDS-PAGE and transferred to PVDF+ membrane (Millipore, Massachusetts, USA), followed by incubation with

a rabbit polyclonal antibody to Calpain 1 (Abcam, Cambridge, UK) and HRP conjugated secondary antibodies (Pierce, Illinois, USA). Immunoreactive bands were visualized using Immobilon™ Western chemiluminescence HRP substrate (Millipore, Massachusetts, USA). Equal loading of protein samples was confirmed by coomassie blue staining of the gel. The blots were scanned; bands were normalized by coomassie and quantitated using GelDoc XR system and Quantity One® software version 4.6.3 (Bio-Rad, California, USA). The data represents the values for five independent experiments.

Data Analyses

Data was analyzed using Statistica software (version 10). Normality of the data was checked using Shapiro – Wilk test. Where data was not found to be normal, non – parametric tests were done. Sample means were tested for significance by t – tests, for normal samples independent by groups, and Mann – Whitney U tests for samples which were not found to be normally distributed.

Supplementary table 1: Table shows significant differences between sensillae types between control and pesticide exposed honey bees sampled from field from repeated measures ANOVA tests. No significant difference is observed between sensillae types in laboratory control and pesticide treated samples.

**Sensillae types**	**Field**				**Laboratory**			
	**Adjusted R^2**	**df**	**F**	**p**	**Adjusted R^2**	**df**	**F**	**p**
**Sensory placodea, deep**	0.96625834	58	1690.58026	0	0.042396	58	3.612126	0.062333
**Sensory placodea, shallow**	0.970398998	58	1935.175749	0	0.000668	58	1.039466	0.312180
**Sensory ampullacea**	0.183635527	58	14.27164089	0.000375194	0.002617	58	1.154804	0.286996
**Sensory coeloconica**	0.813956055	58	259.1293753	0	0.026766	58	2.622615	0.110776
**Sensory basiconica**	0.970573194	58	1946.97463	0	-0.008740	58	0.488780	0.487266
**Sensory campaniforme**	0.071281858	58	5.528424117	0.022129204	0.023847	58	2.441361	0.123614
**Sensory trichodea A**	0.761524879	58	189.4052632	0	0.037407	58	3.292800	0.074757
**Sensory trichodea B1**	0.967511995	58	1758.054876	0	0.002980	58	1.176353	0.282588
**Sensory trichodea B2**	0.854319896	58	346.9969643	0	-0.011680	58	0.318819	0.574495
**Sensory trichodea C**	0.815447851	58	261.6928362	0	0.030584	58	2.861382	0.096096
**Sensory trichodea D**	0.996383775	58	16257.35556	0	-0.016108	58	0.064720	0.800086
**Setae A1, A2**	0.909068538	58	590.8403312	0	-0.002965	58	0.825561	0.367319
**Seta A3**	0.88396232	58	450.4555299	0	0.037863	58	3.321822	0.073522
**Seta B**	0.827371544	58	283.7744758	0	-0.016074	58	0.066646	0.797198

Supplementary table 2: Table showing values obtained from Tukey HSD Test after repeated measures ANOVA test of the 14 sensillae types of the honey bee antennae from control and pesticide populations from field and laboratory.

	Field			Laboratory		
**Sensillae types**	p	MS	df	p	MS	df
**Sensory placodea, deep**	0.00011	17.099	58	0.062333395	133.98	58
**Sensory placodea, shallow**	0.00011	12.743	58	0.312179737	75.25	58
**Sensory ampullacea**	0.000375194	0.06337	58	0.286996083	0.06985	58
**Sensory coeloconica**	0.00011	0.18112	58	0.11077607	0.40334	58
**Sensory basiconica**	0.00011	0.36798	58	0.487266451	6.1838	58
**Sensory campaniforme**	0.022129204	0.3213	58	0.123613984	0.12505	58
**Sensory trichodea A**	0.00011	39.015	58	0.074756666	94.889	58
**Sensory trichodea B1**	0.00011	15.223	58	0.282587577	308.7	58
**Sensory trichodea B2**	0.00011	18.165	58	0.574495312	50.237	58
**Sensory trichodea C**	0.00011	1.0505	58	0.096096436	1.9863	58
**Sensory trichodea D**	0.00011	0.25632	58	0.800085985	58.2	58
**Setae A1, A2**	0.00011	16.386	58	0.367319489	10.587	58
**Seta A3**	0.00011	3.4299	58	0.073522014	9.5815	58
**Seta B**	0.00011	18.432	58	0.797198052	43.573	58

Supplementary figures:

Supplementary figure 1: Figure showing the degree of intensification in the high intensity cropping (HIC) site compared to the low intensity cropping (LIC) site based on earlier report by Chakrabarti et al. 2014. Farmer use of pesticides is plotted on secondary axis. No pesticide residues were detected from LIC soil samples.

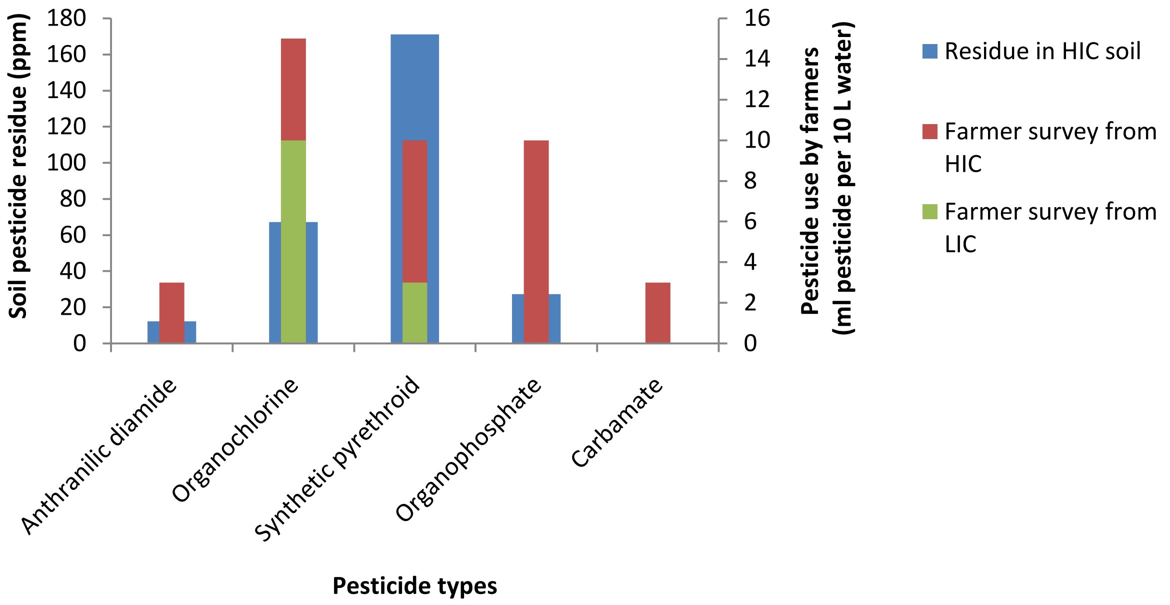

Supplementary figure 2: (a) Sensory basiconica from representative LIC honey bee antenna; (b) Deformed sensory campaniforme from representative HIC honey bee antenna; (c) Segment 4 from a representative HIC honey bee antenna; (d) Segment 4 from a representative LIC honey bee antenna. LIC: Low intensity cropping site; HIC: High intensity cropping site.

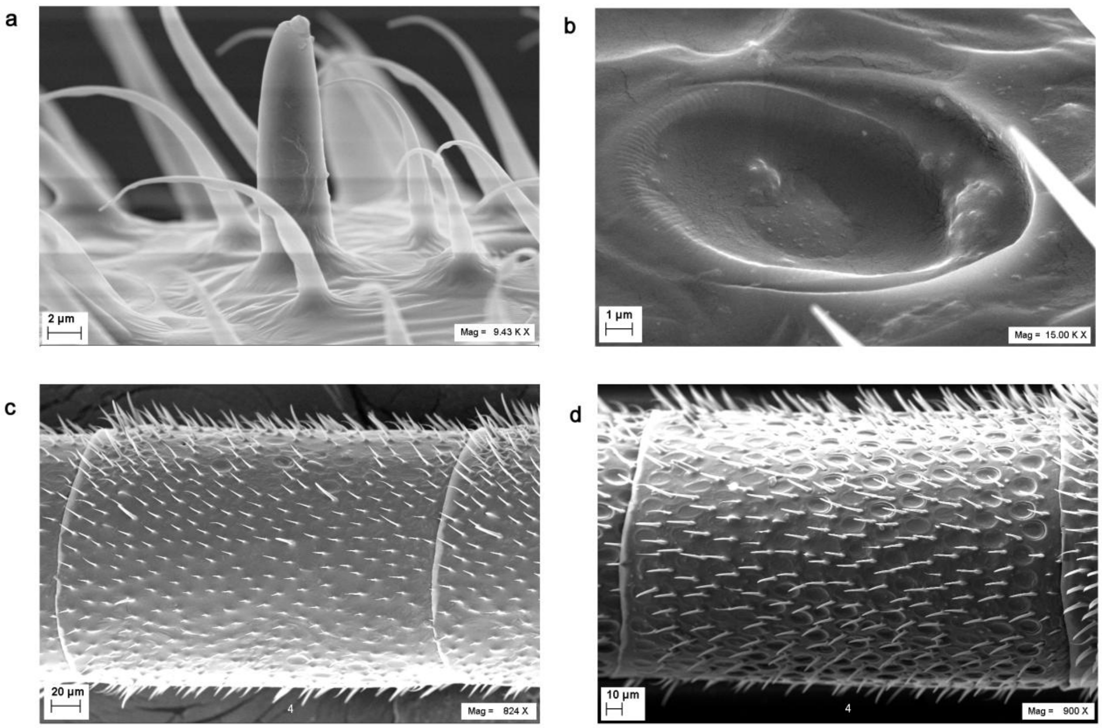

Supplement: supplementary information [file srep12504-s1.pdf]
